# Functional analysis from ex-vivo characterization of LDLR exon 13–15 duplication associated to familial hypercholesterolemia

**DOI:** 10.3389/fendo.2026.1733372

**Published:** 2026-03-06

**Authors:** Catalina Martínez, Carolina Alarcón, Claudia Radojkovic, Andrea Cid, Noemí Vilches, Enrique Guzman-Gutiérrez, Katia Saez, Rodrigo Alonso, Andrea Sánchez

**Affiliations:** 1 Departamento de Bioquímica Clínica e Inmunología, Facultad de Farmacia, Universidad de Concepcion, Concepcion, Chile; 2 Departamento EstadísticaFacultad de Ciencias Físicas y Matemáticas, Universidad de Concepción, Santiago, Chile; 3 Center for Advanced Metabolic Medicine and Nutrition, Santiago, Chile

**Keywords:** *ex vivo* characterization, Exon13_15dup, familial hypercholesterolemia, functional assays, human macrophages

## Abstract

**Background:**

Familial hypercholesterolemia (FH) is an inherited semidominant disorder characterized by high plasma cholesterol levels and increased risk of premature cardiovascular disease. More than 3,000 low-density lipoprotein receptor (LDLR) variants have been identified, most lack functional evidence to determine their pathogenicity. One of them is the exon13_15dup, the most frequent FH-causing variant in Chile. However, its functional impact is poorly understood.

**Objective:**

To determine the functional impact of the exon13_15dup variant in the LDLR, in familial hypercholesterolemia patients.

**Methods:**

Three heterozygous carriers of an exon 13–15 duplication and five wild type subjects were recruited. The peripheral blood mononuclear cells were isolated and differentiated to macrophages. The LDLR expression levels on the cell membrane were evaluated by flow cytometry, subcellular localization by confocal microscopy and LDL incorporation by LDL-FITC uptake assays.

**Results:**

The exon13_15dup variant leads to significantly increased cell-surface LDLR expression and enhanced localization in the endoplasmic reticulum. This results in a reduced capacity for LDL uptake in patient cells, with principal component analysis highlighting distinct differences in LDLR localization compared to wild-type samples.

**Conclusions:**

The functional analysis showed that the mutation affects the proper transport and function of LDLR, resulting in a dysfunctional protein that cannot effectively internalize LDL.

## Introduction

Familial hypercholesterolemia (FH) is a common inherited autosomal semidominant disorder characterized by high plasma cholesterol levels (1–3). Its frequency is estimated to be 1:313 and, if untreated, increases the risk of premature cardiovascular disease (4, 5).

Approximately 90% of FH cases are caused by pathogenic variants in the LDL receptor (LDLR) gene (2, 6). The LDLR in its mature form is a glycosylated protein with a molecular mass of 160 kDa that is expressed the cell surface, where it mediates the uptake of lipoprotein particles from the circulation, mainly LDL (2, 7). Up to now, more than 3,000 variants have been deposited in the ClinVar database. Diverse LDLR variants can impact different phases of LDLR-mediated LDL particle endocytosis, which can be classified into six categories: Class 1 prevents the synthesis of proteins; Class 2 causes partial or total retention in ER; Class 3 impairs apoB apolipoprotein interaction; Class 4 causes impaired endocytosis; Class 5 prevents LDLR recycling to the membrane, and Class 6 variants are incorrectly inserted into the cell membrane (2, 8–10). However, despite the high number of variants identified in clinically diagnosed FH patients, about 85% lack functional evidence to evaluate their pathogenicity (2, 11–13). One of these mutations, with limited functional evidence of pathogenicity, is the duplication of exons 13_15 of the LDLR.

The exon 13–15 duplication (c.(1845 + 1_1846-1)_(2311 + 1_2312-1)dup), denoted exon13_15dup, has been primarily described in Italian population (14). It is located at the EGF- domain of LDL-R and, until now, is the most frequent variant found in Chile (15). The available literature suggests that this mutation would produce a disrupted reading frame and a premature stop codon, which leads to an LDLR protein without the transmembrane domain, incapable of anchored to the plasma membrane (14). Although this variant is a major genetic rearrangement and meets the ACMG and ClinGen criteria to be classified as pathogenic, there is little information regarding its functional effect on *LDLR*.

Determining the pathogenicity of LDLR is a critical challenge in genomic medicine (2). Several approaches, including computer prediction algorithms, *in vivo* and *in vitro* experimental evidence, have been developed to elucidate the impact of genetic mutations (13, 16). In this study, we employed an ex vivo experimental approach to evaluate the expression levels, subcellular localization, and LDL uptake in macrophages differentiated from CD14+ monocytes obtained from patients carrying the exon13_15dup variant. Our goal was to elucidate the functional consequences of this variant, providing functional evidence of its pathogenicity.

## Materials and methods

### Study population and inclusion criteria

Three heterozygous (HeFH) carriers of exon13_15dup and five wild type subjects were recruited. All subjects or their legal guardian gave their informed consent for inclusion before they participated in the study. The research was conducted by the Declaration of Helsinki, and the protocol was approved by the Comité Ético Científico, Servicio de Salud, Concepción (Project Fondecyt Iniciación N°11220497).

### Culture and differentiation of peripheral blood monocytes

Peripheral blood was obtained in EDTA vacutainer collection tubes from all subjects, and peripheral blood mononuclear cells (PBMCs) were isolated from whole blood by Lymphocyte Separation Medium (Corning). Briefly, blood was diluted 1:2 in phosphate-buffered saline (PBS 10 mM) (Cytiva), then 8 mL of diluted blood was slowly layered over 16 of Lymphocyte Separation Medium 1.077-1.080 g/mL (Corning) and centrifuged at 300×g for 20 minutes at room temperature. PBMCs were collected, washed in PBS 10 mM (Cytiva), and resuspended in 80 μL of MACS buffer (PBS 10 mM, 0.5% p/v bovine serum albumin (BSA) (Rockland), and 2 mM EDTA) per 10^7^ total cells. Then, cells were incubated for 15 minutes with 20 μL of CD14 microbeads per 10^7^ total cells (Miltenyi Biotec). Cells were washed and separated with MACS columns, according to the manufacturer’s instructions (Miltenyi Biotec). Monocytes were plated in 6 cm tissue culture dishes at a density of 2×10^6^ cells/mL, using RPMI-1640 supplemented with 2 mM L-glutamine (Cytiva), 100 U/ml penicillin (Gibco), 100 μg/ml streptomycin (Gibco), and 10% fetal bovine serum under standard culture conditions. After 24 hours, cells were supplemented with 25 μg/mL GM-CSF (Sigma), and media were changed every 3 days.

### Quantification of LDLR expression by flow cytometry

To determine LDLR cell surface expression by FACS, 500,000 PBMC-derived macrophages from individuals carrying the exon13_15dup variant and wild-type individuals were resuspended in 200 µL of 10 mM PBS and incubated with 0.002 mg/mL of a BV421-conjugated mouse anti-human LDLR antibody (BD Biosciences). After 30 minutes of incubation at 4°C in the dark, the cells were washed, fixed with IC Fixation Buffer (Invitrogen^®^), and resuspended in 300 µL of 10 mM PBS to be analyzed by flow cytometry. One drop of beads containing 0.002 mg/mL of the BV421 antibody was used as a positive control for the LDLR marker. Anti-human CD14+ mouse IgG antibodies APC/Cyanine7 (Biolegend^®^) and anti-human CD16+ mouse IgG antibody PE/Cyanine7 (Biolegend^®^) were used as a macrophage cell lineage marker.

For each sample, 10,000 events were acquired for data analysis using the FORTESSA-X20 FACS. The results were observed through the FlowJo program.

### Cell localization by immunocytochemistry

CD14+ cells were seeded onto glass coverslips and differentiated into macrophages as previously described. Cells were fixed with 4% paraformaldehyde in 10 mM PBS for 15 minutes and permeabilized with 0.5% Triton X-100 in Tris-phosphate buffer [Na_2_HPO_4_ 0.085 M, KH_2_PO_4_ 0.035 M, NaCl 1.2 M Trisaminomethane (HOCH_2_)_3_CNH_2_ 0.1 M (Tris-PO_4_ buffer)] for 10 minutes at room temperature. The cells were washed with Tris-PO_4_ buffer and incubated overnight at 4°C with the following antibodies: 2 µg/mL goat anti-human LDLR polyclonal antibody (R&D Systems), 20 µg/mL calreticulin and 30 µg/mL lysosomal-associated membrane protein 1 (LAMP1). Then, the cells were incubated at room temperature for 2 hours with Cy3 AffiniPure Donkey Anti-Goat IgG, Alexa Fluor 488 AffiniPure Donkey Anti-Rabbit IgG, or Cy2 AffiniPure Donkey Anti-Mouse IgG. 4′,6-diamidino-2-fenilindol (DAPI) was used for nuclear staining. The images were acquired using an LSM 780 spectral confocal microscope (Zeiss), the intensity profile was determined with ImageJ software.

### Lipoprotein isolation and labeling

Blood plasma for lipoprotein purification was collected from healthy individuals by centrifugation at 12,000×g for 30 minutes at 4°C. LDL (1.019–1.050 g/mL) was isolated by a sequential density gradient centrifugation using KBr for density adjustment. Plasma ultracentrifugation was carried out in a Thermo T-865 rotor (Kontron, Germany) at 38,000 rpm for 20 hours at 15°C in a Centrikon T-21X0. The intermediate orange band corresponding to LDL, was collected dialyzed in buffer PBS 10 mM for 48 hours. Protein was performed using the PierceTM BCA Protien Assay kit (ThermoFisher). LDL was stored in the dark at 4°C, in the presence of an antioxidant (BHT). Isolated lipoproteins were used within 2–3 days after purification.

For LDL labeling, 40 µg of LDL were incubated with fluorescein iso-thiocyanate (FITC), according to the manufacturer’s instructions (FluoReporter™ FITC Protein Labeling Kit, Invitrogen).

### Lipoprotein uptake assay

CD14+ cells were seeded onto sterile glass coverslips at a density of 2x10^6^ to 3x10^6^ cells/mL cells per well. Prior to uptake assays, the medium was removed, and cells were washed with 10 mM PBS and incubated in a lipid-free medium for 4 hours. For the transport assay, cells were incubated for 0 and 15 minutes with 20 μg/mL of (LDLC-FITC). For competition assays, unlabeled LDL was added at a 1:2 and 1:4 molar ratio to LDLC-FITC. Subsequently, the cell membrane was stained with CF594^®^-Wheat Germ Agglutinin (WGA) (Biotium^®^) and cells were fixed with Fixation Buffer (Invitrogen^®^) for 10 minutes at room temperature. Extracellular fluorescence was quenched with 0.2% v/v Trypan Blue. Cells were then permeabilized with 10 mM Tris-PO4 buffer for 10 minutes at room temperature, and nuclei were stained with DAPI (ThermoFisher^®^). As a negative control, CD14+ cells were incubated with 20 μg/mL of unlabeled LDL. Finally, samples and controls were analyzed using a Zeiss LSM 780 spectral multiphoton microscope. Image analysis was performed with ImageJ.

### Statistical analysis

Data analysis was performed using the Student t-test of unpaired data for univariate experiments with a 95% interval (p<0.05) in the GraphPad PRISM statistical software.

## Results

### Cell surface expression and subcellular localization of LDLR in macrophages derived from peripheral blood monocytes

**Figure 1:**
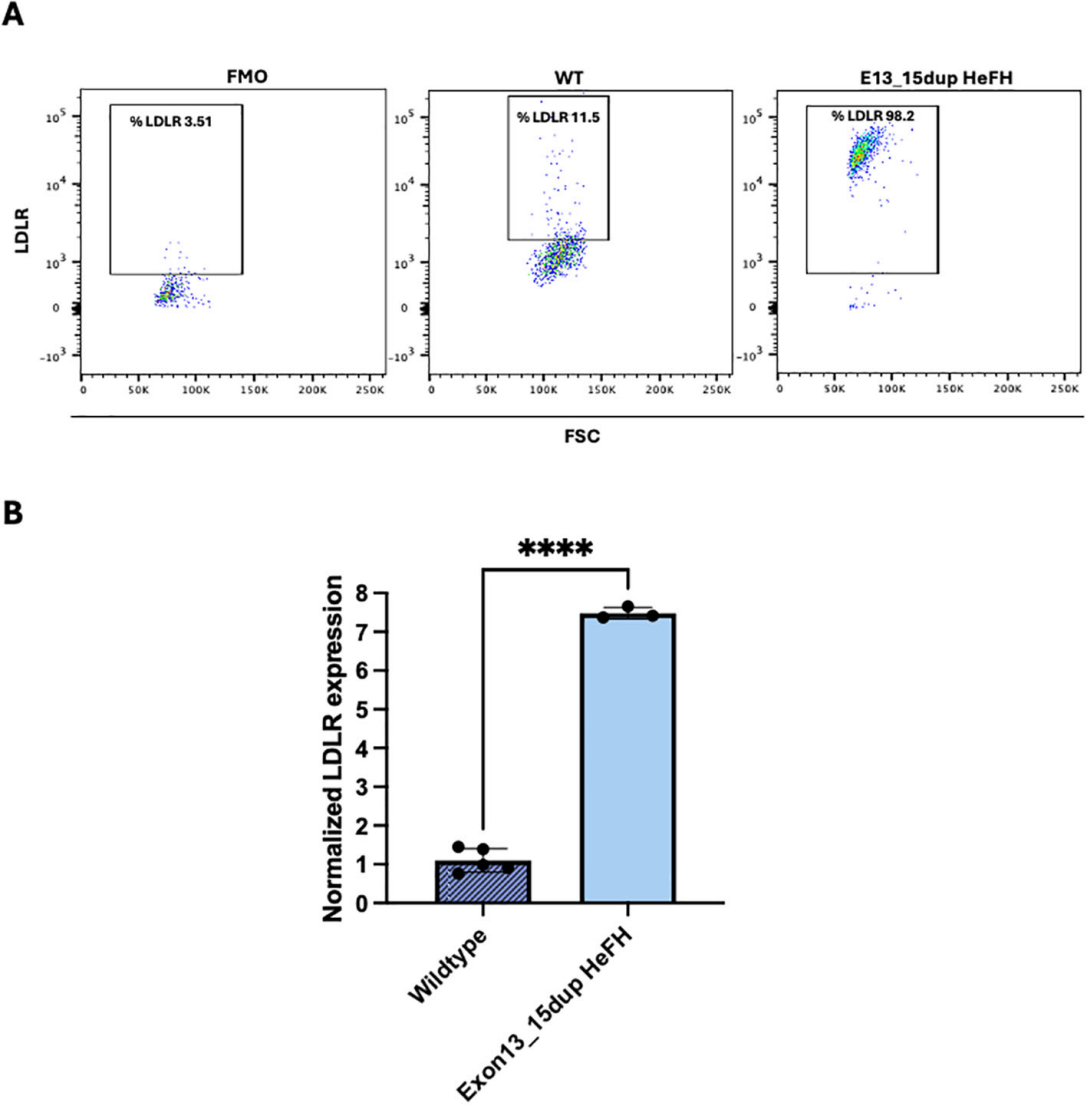
Determination of LDLR expression in PBMC-derived macrophages from wild-type individuals and patients carrying the exon13_15dup. **(A)** Left panel, representative dot plot corresponding to macrophages from FMO control, wild-type subjects and heterozygous carrier of the exon13_15dup variant. **(B)** MFI quantification of the LDLR marker from samples from wild-type individuals (n=7) and patients carrying the exon13_15dup variant (n=3) (****p < 0.0001). FSC, Forward scatter; LDLR, LDL receptor; FMO, LDLR negative labeling; FH, Familial Hypercholesterolemia; He, Heterozygous; FSC, Forward Scatter.

Flow cytometry was used to detect the number of positive LDLR cell-surface expression in macrophages derived from peripheral blood monocytes from exon13_15dup HeFH patients and controls. This analysis revealed that the percentage of LDLR-positive cells was significantly higher in HeFH samples compared to wild-type controls (97.5 ± 2.0 vs. 13.9% ± 2.5, p<0.0001) (**Figure 1**).

**Figure 2:**
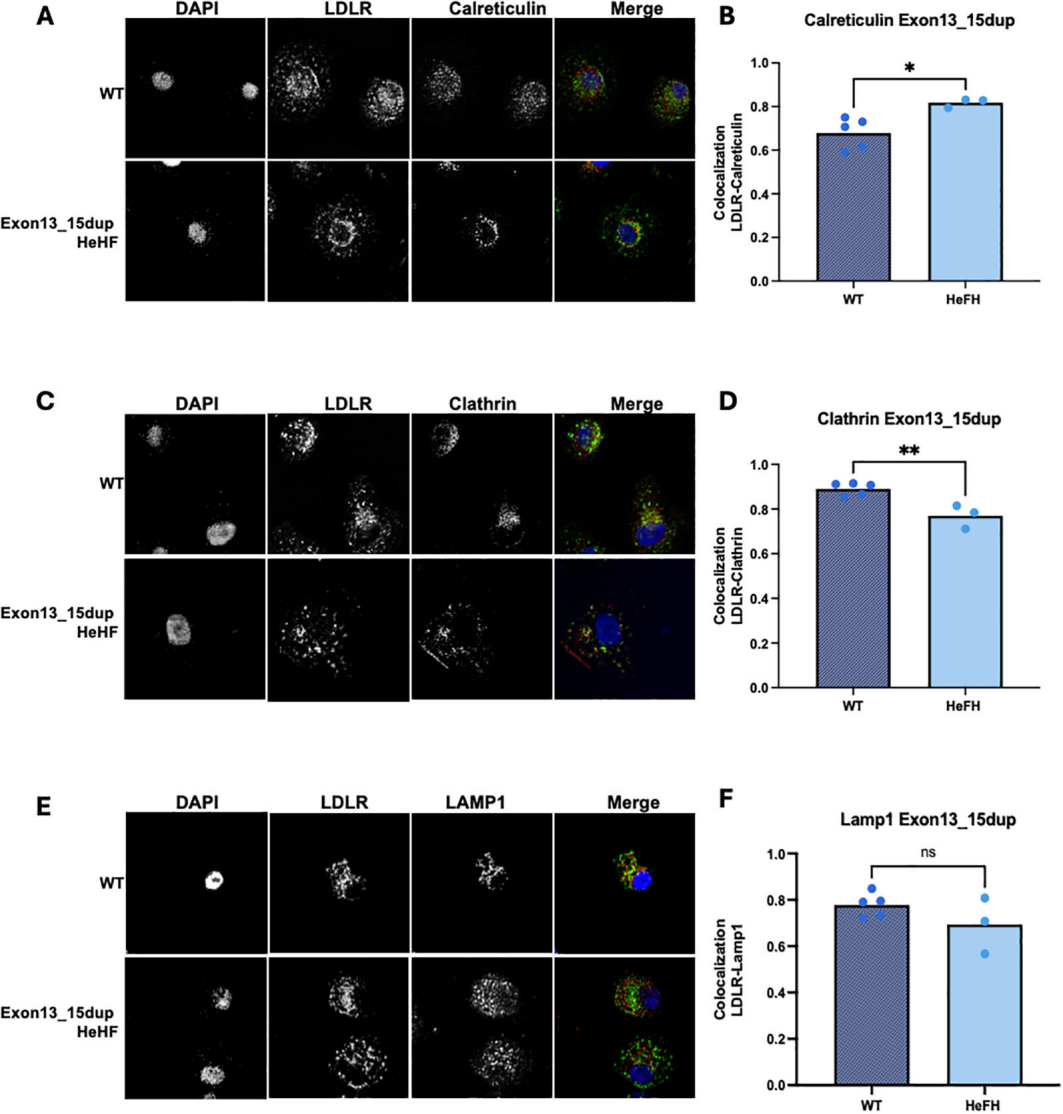
Immunocytochemistry of LDLR and subcelular marker in macrophages derived from PBMC of Exon13_15dup patients. **(A, C, E)** Representative images at 40x magnification of the wild-type and Exon13_15dup genotypes colocalization of LDLR with calreticulin, clathrin and LAMP-1. **(B, D, F)** Quantification of LDLR marker colocalization in samples from wild-type and patients carrying the variant with the subcellular markers. Colocalization was determined by image deconvolution and Pearson compression measurement using ImageJ. Wild type individuals (n=5)) and patients carrying the exon13_15dup variant (n=3). *= p<0.05, **= p<0.01, ns= non significant.

To analyze the effect of the exon13_15dup LDLR variant on cellular localization, confocal microscopy assays were performed using the co-localization of the LDLR with endoplasmic reticulum (Calreticulin), clathrin pits (Clathrin), and lysosomal (Lamp1) markers. Confocal images showed that the exon13_15dup LDLR variant was expressed in patient-derived macrophages showing significantly higher expression levels in the ER, as indicated by the colocalization with calreticulin (0.82 ± 0.02 exon13_15dup LDLR vs. 0.68 ± 0.07 wild type, p=0.022) (**Figures 2A, B**).

Colocalization analysis with clathrin revealed a significant reduction in the presence of the variant (0.77 ± 0.05 exon13_15dup LDLR vs. 0.89 ± 0.03 wildtype, p=0.0053) (**Figures 2C, D**), while no significant differences were observed with the lysosomal marker (0.69 ± 0.12 exon13_15dup LDLR vs. 0.78 ± 0.05 wild-type, p=0.2078) (**Figures 2E, F**). These findings suggest that the exon13_15dup LDLR variant enhances receptor synthesis, potentially leading to decreased receptor internalization. This is evidenced by its significant colocalization with endoplasmic reticulum (ER) markers, elevated levels of membrane expression, and reduced colocalization with endocytosis markers such as clathrin and LAMP1. These results align with the characteristic cytoplasmic dot-like pattern seen in wild-type individuals, representing *de novo* trafficking and active recycling (17), which is notably altered in exon13_15dup cells.

### LDL uptake assays

**Figure 3:**
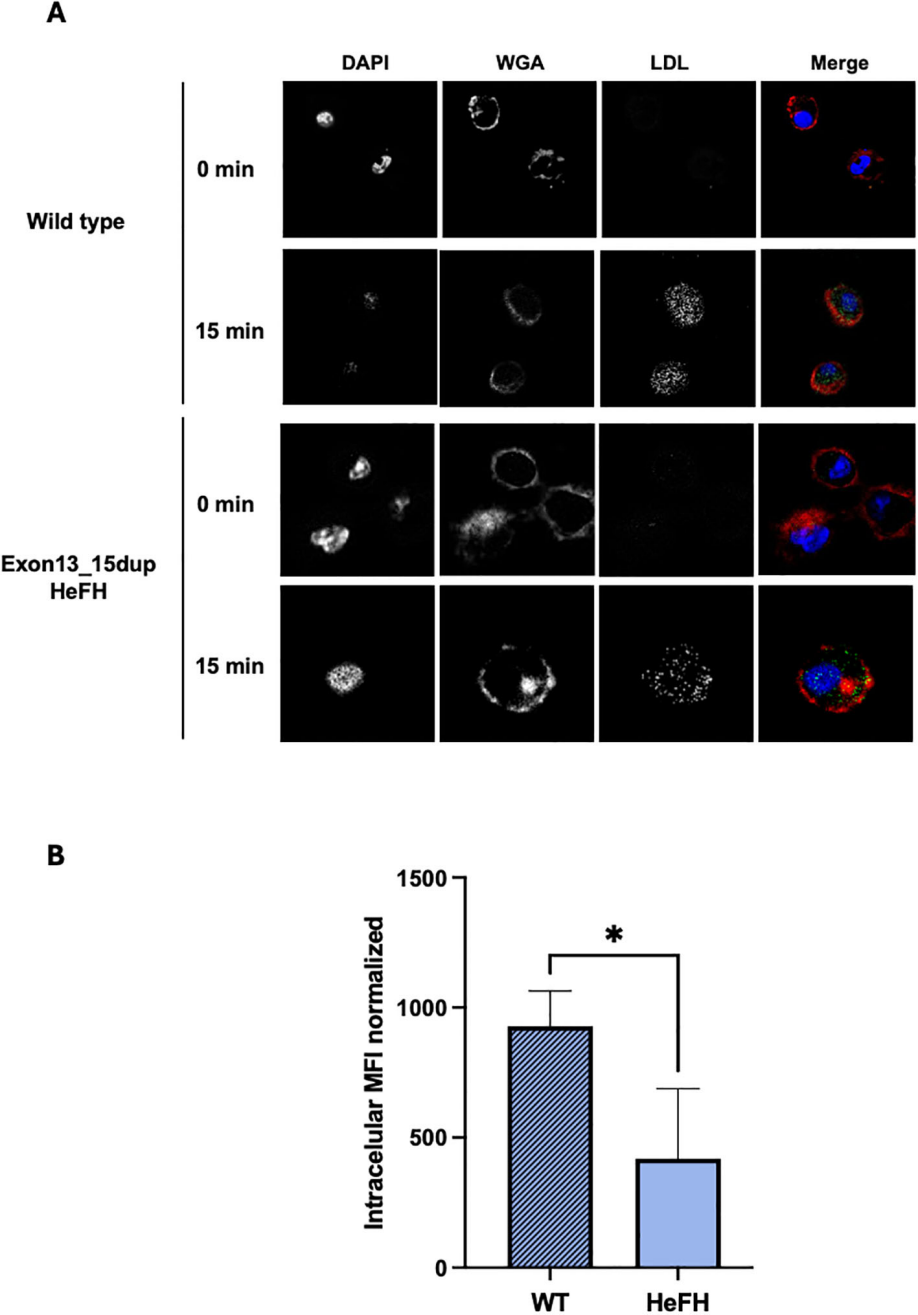
LDL-C transport in macrophages from patients with Exon13_15dup variant. **(A)** Representative confocal images of LDL transport at 0 and 15 minutes by PBMC-derived macrophages in wildtype (upper panel) and Exon13_14dup (bottom panel) subjects. **(B)** Quantification of LDL- FITC into the cell interior of cells from patients carrying the Exon13_15dup variant. Fluorescence quantification was performed using ImageJ in stacked images of 20 planes; these results were normalized by a time of 0 minutes. Wild-type (n=5), exon13_15dup patients (n=3); *p<0.05.

To evaluate the internalization capacity of LDL by the exon13_15dup variant, transport assays with LDL-FITC were performed and analyzed by confocal laser scanning microscopy. The results showed that cells from HeFH patients retain the ability to internalize LDL, albeit with a significantly diminished capacity in 15 minutes in comparison with the wild-type (418.7 ± 269.4 vs. 928 ± 135, p=0,043). These results suggest that the variant alters the kinetics of LDL transport, leading to a slower rate of internalization in the presence of the duplication (**Figure 3**).

## Discussion

Currently, more than 3,000 different variants of the LDLR gene have been described, of which approximately 50% require functional evidence to support their pathogenicity. In this context, functional assays represent a simple method for determining whether the activity of a mutant protein (18). In recent years, accessible methodologies have been developed to evaluate LDLR activity *in vitro*, providing experimental reproducibility between laboratories around the world that ensures rigorous analysis of all functional studies (19). However, ex vivo offers advantages over *in vitro* approaches by enabling the exploration of LDLR activity within the context of other patient-specific proteins involved in LDLR pathways (18). Furthermore, patient cell-based methods, such as those using peripheral blood mononuclear cells, are ideal for high-throughput screening. Their advantages include minimally invasive isolation, rapid stimulation, and their ability to reflect the LDL receptor status of other cell types within the body. In this report, we used PBMC-derived macrophages from FH patients as a study model system. Monocyte-macrophages play a central role in the development of atherosclerosis as they constitute a primary cellular component of innate immunity within atherosclerotic plaques and drive the proinflammatory response (20, 21) complex internal structure with abundant cytoplasm, facilitating a detailed evaluation of LDLR subcellular localization in cells from patients and controls.

The exon13_15dup LDLR variant, designated FH Bologna 2, was originally described by Lelli et al. (13). They proposed that this duplication leads to a protein deficient in the transmembrane domain, a finding subsequently corroborated by sequencing and molecular dynamics (22). Although this variant was identified in 1991, there is still a lack of functional assays to definitively establish its pathogenicity. In this report, we demonstrate that PBMC-derived macrophages from patients carrying this variant exhibit significantly elevated number of positive LDLR cell-surface expression compared to wild-type controls. This finding is consistent with observations for the pathogenic receptor-deficient variant p.(Cys681*), which similarly produces a truncated LDLR protein lacking essential domains for receptor stabilization, membrane anchoring, and internalization (13, 23, 24). For that variant, ex vivo flow cytometry assays showed a 54.3% increase in LDLR expression alongside a ~30% reduction in LDL binding and uptake, suggesting an attenuation of hepatic metabolism and a severe clinical phenotype (13).

Functional assays are a direct method to determine whether the activity of a mutant protein is altered by considering all the involved biological mechanisms (18, 25).

To analyze the cellular impact of the exon13_15dup variant, *ex vivo* functional validation was performed via immunocytochemistry in PBMC-derived macrophages. Our results demonstrated increased colocalization of LDLR with endoplasmic reticulum (ER) markers and decreased colocalization with clathrin in patients carrying the exon13_15dup variant compared to wild-type subjects. These findings suggest a potential pathogenic mechanism (18, 19, 25), wherein the reduced interaction between LDLR and clathrin indicates impaired internalization of the LDL-LDLR complex. This defect likely triggers a compensatory cellular response via transcriptional activation of the sterol regulatory element (SRE)-1 within the *LDLR* promoter (21, 25). This feedback loop leads to increased receptor synthesis, subsequently increasing its localization within the ER. Furthermore, the differential localization of calreticulin observed in macrophages from heterozygous familial hypercholesterolemia (HeFH) carriers versus wild-type individuals may stem from this heightened demand for cholesterol, which drives *LDLR* transcription and translation. The resulting protein localization in ER, combined with potential alterations in folding kinetics for this specific variant, may induce ER stress and alter its steady-state cellular distribution. In wild-type individuals, LDLR typically exhibits a punctate (‘dot-like’) cytoplasmic distribution, reflecting both *de novo* trafficking to the plasma membrane and active receptor recycling. In contrast, the duplicated variant appears to disrupt these trafficking pathways, leading to a distinct expression pattern characterized by a lack of recycling. However, to confirm these findings, further complementary assays are required, such as the quantification of ER stress markers, assessment of SREBP activation, and the biochemical evaluation of mature versus precursor LDLR forms.

The apparent discrepancy between the high frequency of LDLR-positive events observed in flow cytometry and the functional deficits identified via confocal microscopy reflects a complex interplay between cellular compensation and structural dysfunction. The approximately 7-fold increase in the percentage of positive events (indicating a higher number of positive cells expressing LDLR) in patient-derived cells suggests a compensatory upregulation. This phenomenon is likely driven by the SREBP pathway (21); as the exon13_15dup variant is functionally defective, the resulting low intracellular cholesterol levels trigger a feedback loop that increases mRNA and protein synthesis to restore homeostasis. However, our microscopy data reveals that this increased synthesis does not translate into functional cholesterol uptake. The truncated protein, which lacks most of the transmembrane domain and the entire C-terminal NPxY motif (22), leads to significant localization in ER due to a higher synthesis. Furthermore, the fraction of the receptor that manages to reach the plasma membrane lacks both stable anchoring and the ability to interact with clathrin-coated pits. Nevertheless, further studies are warranted to better understand these mechanisms.

In addition to the altered cellular localization of this variant, functional assays demonstrated a significant reduction of 45% in LDL uptake by macrophages-derived PBMCs from patients heterozygous for the exon13_15dup at 15 minutes. Transport times are consistent with those previously reported for this receptor, which have shown that LDL internalization occurs rapidly within 10 minutes and cholesterol ester components are degraded within 60 minutes (26, 27). Altogether, these results are consistent with an impaired endocytic mechanism, characterized by increased receptor expression—supported by higher colocalization in the ER and membrane expression—and decreased colocalization with clathrin, representing the endocytic pathway. The global reduction in intracellular cholesterol due to altered endocytosis would maintain the activation of compensatory mechanisms, including LDLR synthesis.

The functional implications of the LDLR exon13-15dup variant remained largely unexplored until our group’s recent structural characterization (22). While FH-associated variants are primarily linked to impaired LDL clearance, LDLR mutations often exert broader pleiotropic effects on vascular homeostasis, intracellular signaling, and inflammatory responses. Given our previous structural analysis, the functional impact of the exon13-15dup variant likely extends beyond defective endocytosis to include disrupted signaling pathways, such as the PI3K/Akt/mTOR axis, which has been shown to regulate endothelial cell autophagy downstream of LDLR activation (28). Furthermore, the LDLR is known to undergo proteolytic cleavage by ADAM-17 and MMP-14 at the cell surface, generating a soluble form (sLDLR) that impairs LDL-C uptake and correlates with pro-atherogenic profiles, including increased VLDL particles, elevated triglycerides, and a higher risk of myocardial infarction (29, 30). For the exon13-15dup variant, the observed structural changes—particularly a potentially reduced membrane-anchoring capacity—could further promote the generation of sLDLR. This, in turn, would exacerbate disturbances in plasma lipoprotein composition and clearance, while triggering pleiotropic effects across various tissues, including the vascular bed.

The systemic relevance of LDLR dysfunction is further supported by evidence from LDLR KO models, which exhibit impaired arterial distensibility and MCP-1-induced vascular remodeling (31); similar pathological features could theoretically be present in carriers of the exon13-15dup variant. In support of this, clinical data from FH patients treated with inclisiran (a siRNA inhibiting hepatic PCSK9) demonstrated that the reduction in LDL-C levels correlated with improvements in pulse wave velocity, suggesting a beneficial effect on arterial stiffness (32). Collectively, these findings suggest that the exon13-15dup variant may drive a complex pro-inflammatory and pro-atherogenic phenotype mediated by both LDL-C-dependent and independent mechanisms, warranting further investigation.

In summary, our functional evidence regarding protein transport and localization, together with the re-evaluation of this variant according to the ACMG/ClinGen FH VCEP criteria, which meets the evidence for PVS1_Strong, PM2, PS4, PP4, and PP1 (33), justifies reclassifying this variant as pathogenic in clinical databases. These findings provide a molecular explanation for the hypercholesterolemia observed in carriers and facilitate more precise clinical management.

## Data Availability

The original contributions presented in the study are included in the article/supplementary material. Further inquiries can be directed to the corresponding author/s.
